# Evaluation of Antioxidant and Antimicrobial Properties of Murtilla Leaves (*Ugni molinae Turcz*.) in Beef Patties: Effects on Quality Parameters and Shelf Life

**DOI:** 10.3390/foods13244174

**Published:** 2024-12-23

**Authors:** Lidiana Velázquez, John Quiñones, Ailín Martínez, Isabela Pérez, Carla Velasquez, Gastón Sepúlveda-Truan, Rommy Díaz, Paulo Cezar Bastianello Campagnol, Néstor Sepúlveda

**Affiliations:** 1 Center for Meat Technology and Innovation (CTI-Carne), University of La Frontera, Temuco 4780000, Chile; l.velazquez01@ufromail.cl (L.V.); john.quinones@ufrontera.cl (J.Q.); a.martinez26@ufromail.cl (A.M.); i.perez04@ufromail.cl (I.P.); c.velasquez12@ufromail.cl (C.V.); g.sepulveda10@ufromail.cl (G.S.-T.); rommy.diaz@ufrontera.cl (R.D.); 2 Doctoral Program in Agro-Food Sciences and Environment, University of La Frontera, Temuco 4780000, Chile; 3 Department of Agricultural Production, Faculty of Agricultural and Forestry Sciences, University of La Frontera, Temuco 4780000, Chile; 4 Doctoral Program in Sciences with a Mention in Advanced Cellular and Molecular Biology, University of La Frontera, Temuco 4780000, Chile; 5 Department of Technology and Food Science, Federal University of Santa Maria, Santa Maria 97105-900, RS, Brazil; paulo.campagnol@ufsm.br

**Keywords:** *Ugni molinae*, antimicrobial activity, oxidative stability, quality indicators, beef patties

## Abstract

Beef patties are highly consumed worldwide. However, its formulations often include synthetic antioxidants and antimicrobials. Murtilla (*Ugni molinae Turcz*.), a shrub native to southern Chile, has leaves with a polyphenolic concentration 3.2 times higher than its fruits. This study evaluated the effects of three concentrations of murtilla leaf powder (500, 1000, and 1500 mg/kg) on the microbiological, physicochemical, and sensory parameters of beef patties, compared to controls without antioxidants and with erythorbate (500 mg/kg). The patties were stored at 2 °C for 12 days in a modified atmosphere (80% O_2_/20% CO_2_). No changes were observed in proximal composition; however, the 1500 mg/kg concentration affected the redness (a*) of the beef patties (15.04 vs. 19.37 in the control) (*p ≤* 0.05). Oxidative stability increased as follows: Mu1500 (88.21%) > sodium erythorbate (83.5%) > Mu500 (79.7%) > Mu1000 (78.8%). Natural antioxidants decreased the deterioration of essential fatty acids such as linoleic acid. Aerobic mesophilic microorganism growth in the murtilla treatment was lower than in the control (2.06 log cfu/g vs. 3.83 log cfu/g). Murtilla leaf powders show promising results as a substitute for synthetic antioxidants and antimicrobials. Mu500 treatment improved the physicochemical and microbiological quality parameters without compromising the sensory characteristics.

## 1. Introduction

Beef patties are among the most popular processed meat products worldwide due to their affordability, quick preparation, and caloric and nutritional contributions [1,2]. Beef patties have become a staple daily food item not only in Western countries but also in Asian countries, which traditionally have more conservative eating habits [3]. The world’s largest consumer of beef patties is the United States, with an annual consumption of approximately 5 billion [4]. In Chile, beef patty consumption has increased significantly in recent years, particularly among the younger population [5]. This growth has paralleled a growing interest in plant-based alternatives, products with reduced sodium, saturated fats, and functional ingredients [6,7].

Beef patties are highly perishable foods, prone to rapid microbial spoilage and oxidative rancidity [8]. Under refrigeration at 4 °C, they have a shelf life of 1 to 2 days, and in frozen storage, 3 to 4 months [9]. Their nutritional composition, which is high in proteins, minerals, vitamins, and fats, makes them a nutritionally valuable food, but it also presents challenges for preservation. The main degradative processes that affect its stability and quality are microbial deterioration and lipid oxidation [10].

Processed meat products often display various nutritional warning labels or are categorized as unhealthy foods; one of the reasons for this is the use of synthetic antioxidants and preservatives [11,12]. Growing consumer demand for healthier alternatives to these types of meat products caused researchers in the meat sector to look for natural and healthy alternatives for traditional antioxidants and preservatives.

Murtilla *(Ugni molinae Turcz.)* is a shrub endemic to southern Chile. This wild plant has been used for centuries by the indigenous peoples of Chile as part of their traditional medicine to treat various ailments [13,14]. Studies conducted over the last few decades have demonstrated that extracts from its leaves have bioactive activity and prebiotic effects, promoting the growth of bacteria such as *Lactobacillus* and *Bifidobacterium* [13]. Furthermore, murtilla leaves contain high concentrations of total polyphenols (128 ± 6 mg gallic acid/g dry leaves) and a variety of bioactive compounds, such asphenolic acids, flavonols, glycosylated flavanols, and flavonoids [13,15], which contribute to significant antioxidant potential ORAC (280 ± 10 mg Trolox/g), FRAP (192 ± 4 mg Trolox/g), TEAC (286 ± 13 mg Trolox/g), and DPPH (361 ± 13 mg Trolox/g) [16]. A recent study demonstrated that the use of murtilla fruit improves the organoleptic indices of Frankfurt-type sausages [17]. It has also been shown that murtilla leaves have a polyphenolic concentration 3.2 times higher than their fruits and, consequently, a superior antioxidant power [15,16]. Therefore, the present study aimed to evaluate the effects of the addition of 500, 1000, and 1500 mg/kg murtilla leaves on the physicochemical, microbiological, and sensory parameters of beef patties stored at 2 °C for 12 days in a modified atmosphere (80% O_2_/20% CO_2_).

## 2. Materials and Methods

Murtilla leaves, collected in May 2021 from the Maquehue Experimental Field of the Universidad de La Frontera, Chile (38°50′16.01′′ S. 72°41′39.98′′ W), were utilized for the study. The leaves were sanitized with a 10% sodium hypochlorite solution and subsequently dried in an oven at 35 °C (BINDER ED25, Tuttlingen, Germany). Subsequently, the leaves were ground in an ultracentrifugal mill (Retsch model ZM 200, Haan, Germany) to obtain a fine powder of 80 μm. The powders were vacuum-packed in polyethylene bags and stored at −21 °C until further use.

### 2.1. Beef Patties Preparation

A completely randomized block design was used, divided into two batches with five treatments per batch and 25 experimental units per treatment (2 × 5 × 25): (a) control without antioxidants (CON); (b) 500 mg/kg sodium erythorbate (AS); (c) 500 mg/kg of murtilla leaf powder (Mu500); (d) 1000 mg/kg of murtilla leaf powder (Mu1000); and (e) 1500 mg/kg of murtilla leaf powder (Mu1500). Beef (80%) and pork backfat (20%) were ground using 8 mm and 6 mm plates, respectively, with a refrigerated meat grinder (La Minerva, Bologna, Italy). After meat and fat grinding, 1.5% water and 3.75% salt were added to the total mass. Subsequently, it was divided into five equal parts, and sodium erythorbate and the three levels of murtilla leaf powder were incorporated according to the corresponding treatment. Each portion was mixed manually for five minutes to ensure integration and uniform distribution of the ingredients and allowed to rest for one hour at 2 °C. The beef patties (3 units) were then placed on trays containing an absorbent pad, packaged in a modified atmosphere (80% O_2_/20% CO_2_), and stored for 12 days at 2 °C in the dark. A total of 25 units of beef patties of 50 g each, per treatment per batch were prepared. For analyses, three replicates were taken per treatment at each sampling point (0, 4, 7, and 12 d). At each point, pH, color, and lipid oxidation (TBARS) were evaluated. Proximal composition was evaluated at the beginning of storage (day 0), and microbiological analysis was performed on days 0, 8 and 12. Ten beef patties per treatment were separated and stored in vacuum-sealed polyethylene bags at −20 °C for sensory analysis. Additionally, three replicates of beef patties per treatment were freeze-dried for lipid profile evaluation after 12 days of storage at −20 °C. The freeze-drying process was performed in two stages. First, the beef patties were subjected to a rapid freezing process in an IRINOX HC 51/20 (Corbanese, Italy) at −80 °C for 24 h and subsequently freeze-dried (Labconco FreeZone 2.5 L, Kansas City, MO, USA).

### 2.2. Proximate Composition of Beef Patties

The proximate composition of beef patties was determined by analyzing fat content according to the official protocol Am 5–04 [18], as well as moisture [19], protein [20], and ash [21].

### 2.3. pH and Color Parameters

The pH of the samples was determined using a calibrated pH meter (Hanna Instruments Inc., Cluj-Napoca, Romania). A portable colorimeter (Konica Minolta Sensing, Inc., Tokyo, Japan), including the CIELab system, was used to measure redness (a*), yellowness (b*), and lightness (L*). The measurement conditions were as follows: illuminant D65, measuring angle 10°, aperture size 8 mm.

### 2.4. Evaluation of Lipid Oxidation

The concentration of malonaldehyde (MDA) was measured using the TBARS method, described by Vyncke [22], with some modifications. The degree of lipid oxidation was measured via the chromophore compound formed from the reaction between thiobarbituric acid and malonaldehyde. Briefly, two grams of sample was mixed for 10 min with 15 mL of 7.5% trichloroacetic acid (Merck, Germany) containing 0.1% propyl gallate and EDTA. Subsequently, the mixture was filtered, and 5 mL of the filtrate was added to 5 mL of 2-thiobarbituric acid (0.02 M) (Merck, Germany, ≥98%). The solution was then incubated in a boiling water bath for 40 min, cooled, and absorbance was measured at 538 nm. The results were expressed as mg MDA/kg of the sample.

### 2.5. Fatty Acid Profile

Lipids were extracted from the freeze-dried beef patty samples following the methodology proposed by Folch et al. [23] with some modifications. Fatty acid methyl esters (FAME) were formed by adding 1.3 mL of 2 N potassium hydroxide in methanol (Merck, Germany) and 800 µL of n-hexane (Merck, Germany) for each sample. The supernatant was shaken for 30 min. The supernatant was filtered using anhydrous sodium sulfate (0.5 g) (Merck, Germany), and centrifuged at room temperature (2000× *g* for 5 min). Gas chromatography (Clarus 500. Per-kin Elmer, MA, USA) coupled with a flame ionization detector (FID) in split injection mode, and an automatic sampler was used for FAME analysis. An SPTM 2380 fused silica capillary column (60 m × 0.25 mm × 0.2 µm film thickness) (Supelco, PA, USA) was used, and 1 µL of the FAME extract was injected. A gradient program was used where the initial temperature was set at 150 °C, increased at a rate of 1 °C min^−1^ to 168 °C for 11 min, and then increased at 6 °C min^−1^ to 230 °C for 8 min. The detector and injection port temperatures were 250 °C with nitrogen as the carrier gas.

Individual FAMEs were identified via retention time using a standard 37-component FAME Mix C4-C24 (Supelco, PA, USA). The results were expressed in g FA/100 g of the fat.

### 2.6. Aerobic Mesophilic Microorganism Counts

Three replicates per treatment were analyzed on days 0, 8, and 12 of storage. The count of aerobic mesophilic microorganisms was determined according to official guidelines [24]. Ten grams of each sample were homogenized in 90 mL of peptonized solution. Serial dilutions (10^−1^ –10^−5^) were prepared, and aliquots of each dilution were plated on nutrient agar (Sigma-Aldrich, St Louis city, MO, USA). After 48 h of incubation at 30 °C, colonies were counted, considering a valid range of 30 to 300 CFU (<30 insufficient; >300 Unacceptable; N/P Not present).

### 2.7. Sensory Analysis

A trained sensory panel, composed of six people (three women and three men, aged between 34 and 50 years) from the Center for Technology and Innovation in Meat Quality, University of La Frontera, conducted the sensory evaluations focusing on the following attributes: appearance, color, flavor, odor, juiciness, texture, and overall acceptability. The analyses were performed in a white light room with controlled conditions according to the procedures described in NCh-ISO 6658 [25]. Each was cooked on a large S + S/S + S/S + S/S + S double contact electric grill (Milan Toast, Sulbiate, MB, Italy) preheated to 150 °C until a core temperature of 70 °C determined by means of a manipulated probe was reached. A 9-point hedonic scale was used, where nine corresponded to like very much and one to dislike very much.

### 2.8. Statistical Analysis

A completely randomized block design was applied: five treatments × twenty-five patties per treatment × two batches, totaling 250 experimental units which were evaluated. Shapiro–Wilk’s and Levene’s tests were used to assess normality and homogeneity of variance, respectively. Proximate composition, fatty acid profile, and sensory analyses were conducted using one-way ANOVA. The pH, color (a*, b*, and L*), lipid oxidation, and aerobic mesophilic microorganism counts were analyzed via two-way ANOVA, using treatment and storage time as fixed factors. When significant differences were detected in the main factors or their interactions. Duncan’s multiple-range test was applied at a significance level (*p ≤* 0.05) when differences were detected. Results were expressed as the mean ± standard deviation. Statistical analyses were performed using IBM SPSS Statistics 23 software (IBM Corporation, Armonk, NY, USA).

## 3. Results and Discussion

### 3.1. Physicochemical Parameters

The concentration of murtilla leaf powders incorporated in the patties did not alter (*p* > 0.05) their proximate composition (Table 1). The patties’ moisture content ranged from 61.35% to 62.96%, protein from 16.37% to 17.67%, fat from 16.80% to 19.00%, and ash from 2.08% to 2.37% across treatments. These results are consistent with other studies, which reported that incorporating leaf powders from other plant species such as maqui (*Aristotelia chilensis*), lotus (*Nelumbo nucifera*), and barley (*Hordeum vulgare*), at concentrations of 250 to 2000 mg/kg does not alter the proximate composition of beef patties [26,27,28]. However, it has been noted that higher concentrations of murtilla fruit powders (5.3 g/kg) can modify the moisture, fat, and total energy content of Frankfurter-type sausages [16].

pH is one of the main chemical indicators of meat quality [29]. In most species, the final pH of meat ranges between 5.5 and 5.8, which guarantees optimal quality characteristics. Quality declines when the pH exceeds these limits, and all degradative processes are accelerated [30]. This study evaluated the effect of treatment and storage time on the pH of beef patties reformulated with murtilla leaf powders. On the initial day, the pH of the beef patties was between 5.53 and 5.58, with no significant differences during the first days (days 0 and 4) (Table 2). From day 7 onward, significant changes (*p ≤* 0.05) were noted due to the interaction between treatment and storage time. The patties with the synthetic antioxidant sodium erythorbate showed the highest pH (5.31), while the treatment with the highest concentration of murtilla (Mu1500) showed the lowest pH (5.19). This decrease may be attributed to the acidic properties of some polyphenols present in murtilla leaves. The most abundant phenolic compounds in murtilla leaves are myricetin rhamnoside, gallic acid, bis-HHDP-glucose, myricetin hexoside, galloyl-quercetin hexoside, myricetin rhamnoside, quercetin hexoside, myricetin, and quercetin [31]. Lorenzo et al. [32] also reported a further decrease in pH in pork patties reformulated with tea and grape seed extracts stored in a modified atmosphere of 80% O_2_/20% CO_2_. Over the storage period, the total pH reduction ranged from 0.3 to 0.39 units, with specific values for each treatment being: control (0.3), sodium erythorbate (0.32), Mu500 (0.35), Mu1000 (0.36), and Mu1500 (0.39). No significant differences among treatments were observed at the end of the study.

The color indices (a*, b*, and L*) of the beef patties were significantly affected by the combination of treatment and storage time (*p* ≤ 0.05). The inclusion of 1500 mg/kg (Mu1500) of whole murtilla leaf powder with a fine particle size affected the a* redness of the patties from the beginning of storage (15.04) compared to the control (19.37). Similarly, maqui leaf powder at concentrations of 1000 and 2000 mg/kg has also been reported to reduce redness in pork and beef patties [28]. Vegetable leaves possess an intense green color due to chlorophyll, which can affect the bright red color of meat. These results are consistent with those reported by other authors, which indicate that higher concentrations of natural antioxidants impact the color of beef patties [33]. Over the first four days of storage, a reduction of approximately 6 units in the redness index a* was observed in all treatments, and a more pronounced decrease of 14 units (19.37–5.75) was observed between days 4 and 7. According to Lorenzo et al. [32] pork patties are perceived as gray when the redness a* values are between 3.2 and 4.6 and brown when the values are between 4.6 and 10.8. At the end of storage (day 12), patties exhibited low redness and a grayish-brown color, indicative of spoiled products. In general, treatment with sodium erythorbate showed the highest retention of redness a* until day 7, as this synthetic antioxidant inhibits oxidation and acts directly on myoglobin, favoring meat color [34]. The yellowness index (b*) did not show significant differences between treatments, though it decreased during storage. Lightness (L*) increased towards the end of storage, with differences evident between control (65.31) and Mu1500 treatments (55.31).

**Figure 1:**
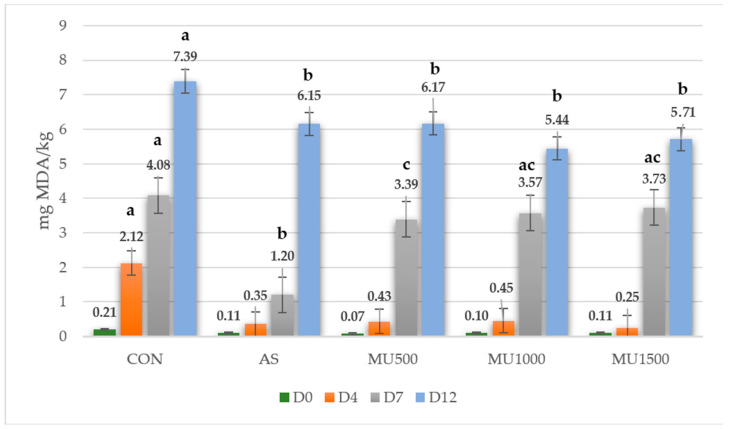
Effects of antioxidant treatment and storage time on lipid oxidation (TBAR value) of reformulated beef patties. CON, control patties; AS, patties with sodium erythorbate; Mu500, patties with 500 mg/kg murtilla leaf powder; Mu1000, patties with 1000 mg/kg murtilla leaf powder; Mu1500, patties with 1500 mg/kg murtilla leaf powder. ^a–c^ Different superscripts indicate significant differences between treatments.

High-oxygen atmosphere packaging (80%O_2_/20%CO_2)_ is commonly used to store beef patties and other minimally processed products, as it maintains myoglobin as oxymyoglobin and promotes the bright red color of meats [35]. However, conditions with high oxygen availability accelerate oxidative processes, reducing the products’ quality and shelf life [36]. Initial lipid oxidation in beef patties was low, but a significant increase in this index was observed from day 4 (*p* ≤ 0.05) (Figure 1), evidencing, a significant interaction between the time of exposure of the beef patties to storage and the treatment (*p* < 0.001). By day 4, the control treatment exceeded the lipid oxidation threshold established for non-rancid products. According to Robles-Martinez et al., [37] lipids can be classified into three categories according to their level of rancidity: non-rancid (TBARS < 1.5 mg MDA/kg), slightly rancid (1.6 < TBARS < 3.6), and rancid (3.7 < TBARS). In contrast, lipid oxidation was significantly (*p* ≤ 0.05) reduced in treatments with sodium erythorbate (83.5%) and murtilla leaf powders Mu500 (79.7%), Mu1000 (78.8%), and Mu1500 (88.21%). The highest concentration of murtilla leaf powder resulted in the lowest oxidation value, suggesting a stronger antioxidant effect. At 7 days, oxidation levels for murtilla leaf powder treatments doubled in relation to day 4, exceeding the acceptable oxidation limit. Nevertheless, oxidation levels were lower than those in the control (*p* ≤ 0.05). A decrease in antioxidant activity of the natural powders was observed on days 4 and 7, likely due to the use of intact powders rather than the purified extracts. Release of polyphenols during storage did not occur in a controlled manner, which would explain why the antioxidant effects did not show a progressive increase but reached a plateau between days 4 and 7. According to Yang et al. [38], the release of phenolic compounds occurs in two stages: an initial burst and a gradual release phase. This is conditioned by factors such as the type of plant material, particle size, structure and morphology, powder aggregation state, and concentration. Shin et al. [39] emphasizes that particle size and structure significantly influence the kinetics of compound release. A smaller particle size increases the contact surface with the dissolution medium, accelerating the diffusion of active compounds into the environment. Therefore, adjusting particle size and optimizing its interaction with the medium could improve the antioxidant effects of plant species when used in the form of powders.

At the end of the storage period, oxidation in Mu1000 and Mu1500 treatments was notably lower (*p* ≤ 0.05) (5.44 and 5.71 mg MDA/kg, respectively). Future studies are needed to evaluate the release kinetics of the extractable and non-extractable fractions in meat matrices when whole-plant powders of murtilla leaves are used instead of their purified extracts.

### 3.2. Fatty Acid Profile

In this study, the fatty acid composition of freeze-dried beef patties was evaluated after 12 days of storage at −20 °C. The fatty acid profiles are presented in Table 3. The distribution of fatty acids in patties showed a predominance of monounsaturated fatty acids (MUFA) (45%) > saturated fatty acids (SFA) (38%) > polyunsaturated fatty acids (PUFA) (17%). Statistical analysis revealed significant differences in the MUFA and PUFA profiles of the beef patties (*p* ≤ 0.05). Mu500, Mu1000, and Mu1500 treatments showed a reduction in MUFA content of 2.26, 1.87, and 1.8%, respectively. In contrast, PUFA content increased by 1.74, 1.21%, and 1.36% for the same treatments.

The reduction in MUFA content was influenced by the levels of oleic acid (C18:1n-9), which were lower in murtilla treatments than in the control (*p* ≤ 0.05). In contrast, linoleic acid (C18:2n-6) concentrations were proportionally higher in beef patties with murtilla powder, which increased by 0.97%, 0.99%, and 1.35% for the Mu500, Mu1000, and Mu1500 treatments, respectively. γ-linolenic acid (C18:3n6) showed significant differences between the control (0.69%) and sodium erythorbate treatments (0.77%) compared to murtilla treatments. In particular, the Mu500 treatment resulted in a higher concentration of this fatty acid (0.93%). Other health-relevant polyunsaturated fatty acids showed significant changes with the inclusion of murtilla leaf powders; eicosapentaenoic acid (C20:5n3) increased in treatments Mu500 and Mu1000, and arachidonic acid (C20:4n6) increased in both natural and synthetic antioxidants (AS). These results allow us to confirm the role of natural antioxidants from murtilla leaves in the quality and oxidative stability of beef patty lipids. The PUFA/MUFA ratio ranged between 0.41 and 0.46 for all treatments. In the treatments with murtilla leaf powder, this ratio was slightly higher (*p* ≤ 0.05).

### 3.3. Microbiological Analysis

**Figure 2:**
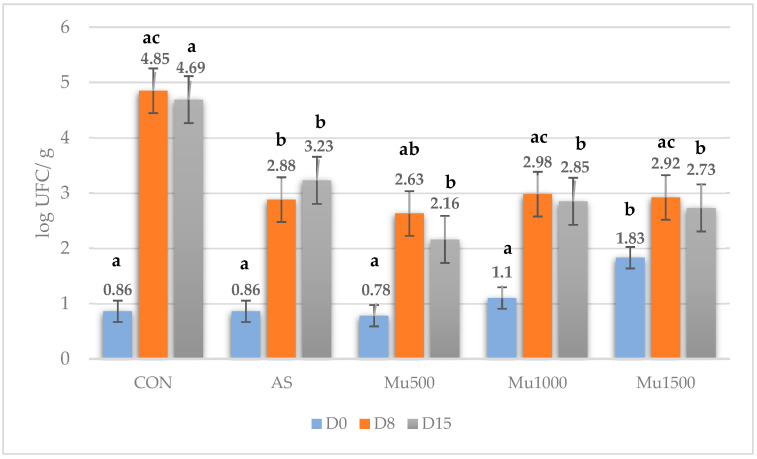
Effects of treatment and storage time on aerobic mesophilic microorganism counts in burgers reformulated with sodium erythorbate and murtilla leaf powder. CON, control beef patties; AS, beef patties with sodium erythorbate; Mu500, beef patties with 500 mg/kg murtilla leaf powder; Mu1000, patties with 1000 mg/kg murtilla leaf powder; Mu1500, patties with 1500 mg/kg murtilla leaf powder. ^a–c^ Different superscripts indicate significant differences between treatments.

Figure 2 shows the aerobic mesophilic counts of patties formulated with murtilla leaf powder over 12 days of storage. Initial counts ranged from 0.86–1.83 log cfu/g, with higher values noted for the Mu1500 treatment (*p* ≤ 0.05). The initial microbial count was considerably lower than that reported in minced beef by other authors (5–6.5 log cfu/g) [40,41], suggesting proper raw material handling during processing. During storage, all treatments showed a significant increase in aerobic mesophilic counts, with an exponential phase of microbial growth between days 0 and 8. A significant interaction (*p* < 0.001) between storage time and treatment was observed, as evidenced by the different microbial growth patterns between the treatments. By day 8, the control treatment exhibited significant (*p* ≤ 0.05) growth of four log units (4.69 log cfu/g), followed by more moderate growth through day 12. Overall, microbial growth of the control treatment throughout storage was 3.83 log cfu/g. In contrast, treatments Mu500, Mu1000, and Mu1500, showed a significantly lower increase (*p* ≤ 0.05) during the exponential phase (1–85–1.91 cfu/g), and an overall increase during storage of 2.06 log cfu/g, indicating microbial inhibition compared to the control. The Mu500 treatment yielded the lowest microbial growth by the end of storage. Polyphenolic extracts from murtilla leaves have shown potent antimicrobial activity against various pathogens, including *Echerichia coli, Listeria monocytogenes, Salmonella enterica, Bacillus subtilis, Micrococcus luteus, Staphilococcus aureus and Staphilococcus epidermidis, Pseudomona aeruginosa, Enterobacter aerogenes,* and against yeasts such as *Candida. albicans* [12,15,42,43,44]. This antimicrobial activity is attributed to compounds such as myricetin, quercetin glucosides, rhamnosides, and xylosides [12,15,42,43,44].

These findings are consistent with other studies that have evaluated the antimicrobial effects of natural extracts in model meat products. Shalaby et al. [40] reported a reduction in the total aerobic bacterial count in minced meat by increasing the concentration of olive leaf extracts (1.2 and 3 mL, corresponding to 35, 70, and 150 mg of phenolic compounds). Similarly, Ahn et al. [45] observed that the inclusion of 1 and 2% beet (*Raphanus sativus*) leaf and root powders significantly reduced microbial counts (approximately 6.4 log cfu/g) compared to the control (7 log cfu/g) in pork patties stored for 14 days. Similarly, extracts obtained from artichoke (*Cynara scolymus* L.) by-products can inhibit the growth of pathogenic bacteria (*E. coli and L. monocytogenes*) during the refrigerated storage of beef patties [46].

### 3.4. Sensory Analysis

**Figure 3:**
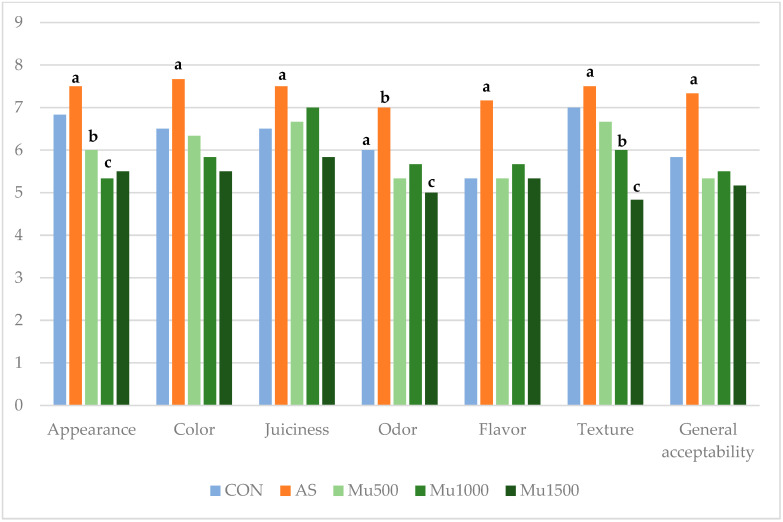
Effect of treatment on organoleptic characteristics of beef patties reformulated with sodium erythorbate and murtilla leaf powder. CON: control beef patties; AS: beef patties with sodium erythorbate; Mu500: beef patties with 500 mg/kg murtilla leaf powder; Mu1000: patties with 1000 mg/kg murtilla leaf powder; Mu1500: patties with 1500 mg/kg murtilla leaf powder. ^a–c^ Different superscripts indicate significant differences between treatments.

The tasters observed no changes in the sensory characteristics of juiciness, odor, flavor, texture, and overall acceptability with the inclusion of murtilla leaf powder (Figure 3). The sodium erythorbate treatment received the best scores for all attributes studied. Appearance and texture was the most differentiated characteristic (*p* ≤ 0.05). Scores for appearance and texture decreased in treatments reformulated with murtilla leaf powder (Mu500, Mu1000, and Mu1500). Among the murtilla concentrations evaluated, Mu500 (500 mg/kg) had the least impact on appearance and texture, whereas the Mu1500 treatment had the lowest scores. For color, patties treated with the synthetic antioxidant sodium erythorbate scored higher (7.6) than other treatments. Sodium erythorbate, used as a synthetic antioxidant, also enhances the color of meat products [34]. It is important to note that no significant differences were found between the control and treatments with murtilla leaf powder, suggesting that these can be incorporated without affecting most of the organoleptic attributes. The 500 mg/kg concentration represents the most favorable in terms of the appearance, while scores for color were not different from those of the control (*p* > 0.05). Furthermore, overall acceptability was not compromised by the addition of murtilla powder, regardless of the concentration used.

## 4. Conclusions

The results of this study indicate that murtilla leaf powders exhibited good antioxidant and antimicrobial activity in beef patties, suggesting that they could be a good substitute for synthetic antioxidants and preservatives for the meat industry. Furthermore, by reducing the oxidation of polyunsaturated fatty acids such as linoleic acid, γ-linolenic acid, eicosapentaenoic acid, and arachidonic acid, these natural extracts have a significant effect on the protection of the nutritional profile of beef patties. Additionally, the antimicrobial effect of murtilla leaf powder enhanced the safety of beef patties by reducing microbial growth during refrigerated storage. Based on these results, a concentration of 500 mg/kg appears to be optimal, allowing the right balance between quality improvement and preservation of organoleptic characteristics. Nevertheless, future studies should be carried out to predict the shelf life of reformulated beef patties through kinetic models and studies evaluating the release kinetics of antioxidant and antimicrobial compounds in the meat matrix. Additionally, market studies should be conducted to evaluate consumer acceptance of beef patties reformulated with murtilla leaf powder.

## Figures and Tables

**Table 1:** Proximal composition of patties formulated with murtilla leaf powders.

Treatment	Moisture (%) *	Fat (%)	Protein (%)	Ash (%)
CON	62.96 ± 0.35	16.80 ± 0.51	17.48 ± 0.28	2.08 ± 0.14
AS	62.41 ± 0.72	17.88 ± 1.65	16.37 ± 1.19	2.29 ± 0.02
Mu500	62.07 ± 0.71	17.46 ± 0.83	17.67 ± 0.36	2.37 ± 0.16
Mu1000	62.07 ± 0.71	17.46 ± 0.83	17.67 ± 0.36	2.37 ± 0.16
Mu1500	61.35 ± 0.72	19.0 ± 1.14	16.56 ± 0.85	2.15 ± 0.16
Sig	n.s.	n.s.	n.s	n.s.

* Mean values ± standard deviation. CON (patties without antioxidant); AS (patties with synthetic antioxidant sodium erythorbate); Mu500 (patties with 500 mg/kg murtilla leaves); Mu1000 (patties with 1000 mg/kg murtilla leaves); and Mu1500 (patties with 1500 mg/kg murtilla leaves). Sig (Significance): n.s. (not significant).

**Table 2:** Effects of murtilla leaf powder inclusion on pH and color indices of beef patties.

Parameters	Treatment	Storage Time				*p*-Value		
		Day 0	Day 4	Day 7	Day 12	AO	T	AO × T
pH	CON *	5.55 ± 0.00 ^1^	5.32 ± 0.00 ^a2^	5.25 ± 0.03 ^ab2^	5.25 ± 0.06 ^2^	0.036	0.000	0.000
	AS	5.53 ± 0.00 ^1^	5.38 ± 0.01 ^2^	5.31 ± 0.02 ^b3^	5.21 ± 0.04 ^4^			
	Mu500	5.58 ± 0.00 ^1^	5.34 ± 0.01 ^2^	5.21 ± 0.04 ^ac3^	5.23 ± 0.04 ^3^			
	Mu1000	5.58 ± 0.00 ^1^	5.41 ± 0.11 ^2^	5.25 ± 0.02 ^ab2^	5.26 ± 0.09 ^2^			
	Mu1500	5.57 ± 0.00 ^1^	5.42 ± 0.12 ^2^	5.18 ± 0.02 ^c2^	5.19 ± 0.06 ^2^			
Color								
a*	CON	19.37 ± 1.16 ^1^	13.20 ± 2.54 ^2^	5.43 ± 0.24 ^3^	2.50 ± 1.10 ^a3^	0.001	0.000	0.000
	AS	17.98 ± 1.55 ^1^	11.12 ± 0.62 ^2^	9.18 ± 0.34 ^a2^	4.47 ± 0.46 ^b3^			
	Mu500	17.41 ± 0.83 ^1^	12.35 ± 0.47 ^2^	5.35 ± 0.83 ^3^	3.05 ± 0.57 ^b4^			
	Mu1000	17.41 ± 0.83 ^1^	13.02 ± 2.13 ^2^	5.75 ± 0.21 ^3^	2.89 ± 0.11 ^b4^			
	Mu1500	15.04 ± 1.64 ^a1^	13.66 ± 2.46 ^1^	5.28 ± 0.25 ^2^	2.96 ± 0.47 ^b3^			
b*	CON	20.84 ± 0.35 ^1^	16.66 ± 0.96	17.36 ± 0.24	16.63 ± 3.01	0.324	0.000	0.069
	AS	19.55 ± 0.45 ^1^	15.38 ± 0.87	17.54 ± 1.22	15.83 ± 1.04			
	Mu500	20.06 ± 1.17 ^1^	17.81 ± 0.89	16.19 ± 0.74 ^2^	17.79 ± 1.19			
	Mu1000	20.06 ± 1.17 ^1^	17.92 ± 1.52	16.04 ± 0.65 ^2^	16.83 ± 0.94			
	Mu1500	19.35 ± 0.85 ^1^	18.41 ± 1.20 ^b1^	16.08 ± 0.95 ^2^	17.04 ± 0.59 ^1^			
L*	CON	52.26 ± 0.78 ^1^	49.98 ± 4.63 ^1^	56.77 ± 0.58 ^a1^	65.31 ± 4.21 ^a2^	0.001	0.000	0.007
	AS	52.29 ± 2.46	50.76 ± 4.24	52.41 ± 1.46 ^b^	56.34 ± 0.59			
	Mu500	53.94 ± 1.86 ^1^	53.37 ± 0.81 ^1^	55.44 ± 1.63 ^ab2^	60.62 ± 3.56 ^2^			
	Mu1000	53.94 ± 1.86 ^1^	52.63 ± 1.39 ^1^	54.46 ± 0.42 ^ab1^	57.32 ± 3.10 ^2^			
	Mu1500	54.18 ± 1.75	51.52 ± 1.13	52.43 ± 1.17 ^ab^	55.31 ± 0.38 ^b^			

* CON: patties without antioxidants; AS: patties with sodium erythorbate; Mu500: patties with 500 mg/kg murtilla leaf powder; Mu1000: patties with 1000 mg/kg murtilla leaf powder; Mu1500: patties with 1500 mg/kg murtilla leaf powder. AO: antioxidant; T: storage time; A × T: interaction factors antioxidant and storage time. a–c Different superscripts in the same row indicate significant differences (effect of treatment; *p* ≤ 0.05); 1–4 different superscripts in the same row (treatment) indicate significant differences between days (effect of storage time; *p* ≤ 0.05).

**Table 3:** Fatty acid profile of beef patties treated with sodium erythorbate and different concentrations of murtilla leaf powder.

(g/100 g)	CON *	AS	Mu500	Mu1000	Mu1500	*p*-Value
**C14:0**	1.48 ± 0.06	1.48 ± 0.02	1.53 ± 0.15	1.55 ± 0.09	1.46 ± 0.06	0.818
**C16:0**	22.61 ± 0.07	22.53 ± 0.23	22.51 ± 0.17	22.59 ± 0.03	22.96 ± 0.07	0.096
**C16:1**	2.34 ± 0.04	2.20 ± 0.10	2.20 ± 0.03	2.22 ± 0.04	2.29 ± 0.05	0.200
**C17:0**	0.78 ± 0.05	0.89 ± 0.07	0.92 ± 0.20	0.85 ± 0.02	0.85 ± 0.04	0.684
**C17:1**	0.65 ± 0.03 ^a^	0.90 ± 0.02 ^b^	0.75 ± 0.08 ^ac^	0.84 ± 0.01 ^bcd^	0.73 ± 0.02 ^c^	0.009
**C18:0**	12.40 ± 0.11	12.67 ± 0.16	12.56 ± 0.04	12.83 ± 0.16	12.50 ± 0.12	0.103
**C18:1n-9c**	41.85 ± 0.23 ^a^	41.24 ± 1.01 ^ab^	39.41 ± 0.12 ^b^	39.74 ± 0.27 ^b^	39.89 ± 0.59 ^b^	0.025
**C18:2n-6t**	1.69 ± 0.03 ^a^	1.68 ± 0.07 ^a^	1.88 ± 0.02 ^b^	1.74 ± 0.04 ^ab^	1.74 ± 0.04 ^ab^	0.027
**C18:2n-6c**	12.41 ± 0.06 ^a^	12.21 ± 0.10 ^a^	13.38 ± 0.22 ^b^	13.40 ± 0.05 ^b^	13.76 ± 0.03 ^b^	0.000
**C18:3n-6**	0.69 ± 0.01 ^a^	0.77 ± 0.01 ^b^	0.93 ± 0.00 ^c^	0.78 ± 0.03 ^b^	0.75 ± 0.03 ^b^	0.000
**C20:0**	0.93 ± 0.03	0.93 ± 0.06	1.02 ± 0.06	0.96 ± 0.13	0.84 ± 0.09	0.370
**C18:3n-3**	0.77 ± 0.03	0.72 ± 0.03	0.81 ± 0.13	0.72 ± 0.04	0.69 ± 0.03	0.457
**C20:1n9**	0.08± 0.11	0.20 ± 0.00	0.30 ± 0.09	0.19 ± 0.01	0.17 ± 0.04	0.149
**C20:4n6**	0.33 ± 0.00 ^a^	0.45 ± 0.01 ^b^	0.44 ± 0.030 ^b^	0.38 ± 0.06 ^a^	0.37 ± 0.04 ^a^	0.046
**C20:5n3**	0.23 ± 0.05 ^abc^	0.20 ± 0.01 ^bc^	0.30 ± 0.00 ^a^	0.27 ± 0.01 ^ab^	0.17 ± 0.01 ^c^	0.014
**C22:6n3**	0.08 ± 0.00	0.14 ± 0.07	0.19 ± 0.07	0.11 ± 0.01	0.06 ± 0.00	0.188
**SFA**	38.23 ± 0.03	39.03 ± 0.59	39.22 ± 0.13	39.38 ± 0.23	39.15 ± 0.28	0.117
**MUFA**	45.10 ± 0.28 ^a^	44.79 ± 0.87 ^a^	42.84 ± 0.00 ^bc^	43.23 ± 0.29 ^bc^	43.30 ± 0.46 ^bc^	0.016
**PUFA**	16.19 ± 0.02 ^a^	16.18 ± 0.29 ^a^	17.93 ± 0.12 ^b^	17.40 ± 0.06 ^b^	17.56 ± 0.18 ^b^	0.000
**trans**	1.69 ± 0.03	1.68 ± 0.07	1.88 ± 0.02 ^a^	1.74 ± 0.04	1.74 ± 0.04	0.027
**PUFA/MUFA**	0.42 ± 0.00 ^a^	0.41 ± 0.00 ^a^	0.46 ± 0.00 ^b^	0.44 ± 0.00 ^c^	0.45 ± 0.00 ^c^	0.000

* Mean values ± standard deviation. *CON: patties without antioxidants; AS: patties with sodium erythorbate; Mu500: patties with 500 mg/kg murtilla leaf powder; Mu1000: patties with 1000 mg/kg murtilla leaf powder; Mu1500: patties with 1500 mg/kg murtilla leaf powder. a–c Different superscripts in the same row and for the same parameter indicate significant differences (treatment effect; *p* ≤ 0.05; Tukey’s test).

## Data Availability

The data are contained in the article.
